# Salient features of mesenchymal stem cells—implications for Ewing sarcoma modeling

**DOI:** 10.3389/fonc.2013.00024

**Published:** 2013-02-25

**Authors:** Michael J. Monument, Nicholas M. Bernthal, R. Lor Randall

**Affiliations:** Sarcoma Services, Department of Orthopaedic Surgery, Huntsman Cancer Institute, University of UtahSalt Lake City, UT, USA

**Keywords:** mesenchymal stem cells, Ewing sarcoma, models, molecular, EWS-FLI1, stem cells

## Abstract

Despite a heightened appreciation of the many defining molecular aberrations in Ewing sarcoma, the cooperative genetic environment and permissive cell of origin essential for EWS/ETS-mediated oncogenesis remain elusive. Consequently, inducible animal and *in vitro* models of Ewing sarcoma from a native cellular context are unable to fully recapitulate malignant transformation. Despite these shortcomings, human, and murine mesenchymal stem cells (MSCs) are the closest working *in vitro* systems available. MSCs are tolerant of ectopic EWS/FLI expression, which is accompanied by a molecular signature most similar to Ewing sarcoma. Whether MSCs are the elusive cell of origin or simply a tolerant platform of the EWS/FLI transcriptome, these cells have become an excellent molecular tool to investigate and manipulate oncogenesis in Ewing sarcoma. Our understanding of the biological complexity and heterogeneity of human MSCs (hMSCs) has increased substantially over time and as such, appreciation and utilization of these salient complexities may greatly enhance the efficient use of these cells as surrogate models for Ewing sarcoma tumorigenesis.

## Introduction

Ewing sarcoma is an aggressive translocation-derived malignancy primarily affecting adolescents and young adults. Balanced chromosomal rearrangements fusing the transcriptional activating domain of the *EWSR1* (Ewing sarcoma breakpoint region 1) gene in frame to the carboxy-terminal DNA binding domain of one of five ETS (E-26) family transcription factors are observed in virtually all tumors (reviewed in Sankar and Lessnick, 2011). EWS/FLI fusions are most common, present in 85–90% of tumors (Delattre et al., 1994; Janknecht, 2005; Sankar and Lessnick, 2011). The EWS/FLI chimera functions as an aberrant oncogenic transcription factor, dysregulating numerous down-stream gene targets (May et al., 1993a,b). Genome-wide sequencing efforts have reproducibly defined a comprehensive EWS/FLI transcriptional signature, characterized by both up- and down-regulated gene targets (Prieur et al., 2004; Kinsey et al., 2006; Smith et al., 2006; Gangwal et al., 2008; Patel et al., 2012). Gene silencing using RNAi-mediated technologies suggests oncogenesis in Ewing sarcoma is dependent on EWS/FLI expression (Prieur et al., 2004; Kinsey et al., 2006; Smith et al., 2006). Otherwise, these tumors are relatively genomically stable and as such, EWS/FLI and related EWS/ETS fusions are felt to be the master regulators of oncogenesis in Ewing sarcoma. This relationship provides a unique opportunity to further understand the precise molecular events responsible for Ewing sarcoma susceptibility and pathogenesis. Detailed in this review, in the absence of an inducible *in vitro* or *in vivo* model of transformation in Ewing sarcoma, multipotent mesenchymal stromal cells [a.k.a. mesenchymal stem cells (MSCs)] have emerged as not only the most likely candidate cell of origin, but provide a workable cellular platform to which EWS/FLI-mediated transformation can be more thoroughly characterized and understood. MSCs are an enigmatic population of cells; our understanding of the biological complexity of these cells continues to evolve, as do techniques to harvest these cells from a variety of anatomical sources. As such, a comprehensive understanding of the salient features of MSC isolation, characterization, and biological attributes is warranted.

## Histogenesis in ewing sarcoma

Unfortunately, despite the inferred hierarchical simplicity of EWS/FLI-mediated gene dysregulation in Ewing sarcoma, *in vitro* and animal models have been unable to fully recapitulate the process of oncogenic transformation under conditions of induced EWS/FLI expression in normal cells and tissues. These challenges, in part stem from an inability to define the precise histogenesis of Ewing sarcoma. Histologically, Ewing sarcoma is an undifferentiated small round blue cell tumor and in contrast to many other cancer models, a direct lineage-specific differentiation of Ewing sarcoma has not been proven. For example, ectopic expression of EWS/FLI in a variety of non-malignant human and animal cell lines is either toxic (Lessnick et al., 2002), induces a gene signature discordant with that observed in patient-derived cell lines (Braunreiter et al., 2006) or is simply incapable of mediating the entire phenotypic spectrum of oncogenic transformation (Lessnick et al., 2002; Riggi et al., 2008). Although beyond the scope of this review, the two most likely “cell of origin” candidates in Ewing sarcoma are neural crest stem cells (NCSC) and mesenchymal progenitor cells/mesenchymal stem cells (MPC/MSC). Supporting a neuroectodermal lineage is that ectopic EWS/FLI expression is tolerated in NCSC (von Levetzow et al., 2011) and gene expression profiles and cell surface antigens in Ewing sarcoma are commonly expressed in neural tissues and NCSCs (Lipinski et al., 1987; Staege et al., 2004; von Levetzow et al., 2011). Conversely, given the predominant skeletal location of primary tumors, a presumed mesenchymal progenitor origin seems likely and indeed, a growing body of literature supports a mesenchymal origin of Ewing sarcoma. Ectopic EWS/FLI expression is tolerated in both human and mouse-derive mesenchymal stem cells (Riggi et al., 2005, 2008; Miyagawa et al., 2008) and the subsequent EWS/FLI-mediated gene signature closely parallels that observed in patient-derived cell lines and primary tumor samples (Miyagawa et al., 2008; Riggi et al., 2008). Alternatively, RNAi-mediated repression of EWS/FLI in patient-derived cells lines results in a reprogrammed genetic profile similar to that of mesenchymal stem cells (Tirode et al., 2007; Potikyan et al., 2008; Kauer et al., 2009). Interestingly, there is mounting evidence advocating that human MSCs (hMSCs) are derived from neuroepithelium via a neural crest intermediate (Takashima et al., 2007; Mendez-Ferrer et al., 2010), suggesting the true cell of origin in Ewing sarcoma may in fact reside along a continuum of the NCSC-MSC progenitor lineage.

Importantly, a global skepticism of these aforementioned cell progenitor models of Ewing sarcoma is that ectopic EWS/FLI expression in either mesenchymal or neural crest progenitors has been unable to consistently recapitulate the full phenotypic spectrum of oncogenic transformation. This maturing evidence suggests a mere permissive cell environment may be insufficient for transformation, prompting speculation that in addition to EWS/ETS gene fusions, other mutations, epigenetic phenomena, or cell cycle modifications are cooperatively necessary in Ewing sarcoma.

## Modeling of ewing sarcoma using mesenchymal stem cells

Numerous cell types have been manipulated in hope to model the induction of EWS/ETS-mediated oncogenesis, including human rhabdomyosarcoma cells, human neuroblastoma cells, mouse and human MPC, NIH3T3 fibroblasts, and human telomerase reverse transcriptase (hTERT)-immortalized human fibroblasts (Lessnick et al., 2002; Rorie et al., 2004; Hu-Lieskovan et al., 2005; Riggi et al., 2005, 2008). Given the highly undifferentiated state of Ewing sarcoma cells, the predominant skeletal location of these tumors, and the expansive anatomic abundance of MSCs, it is of no surprise reports characterizing ectopic EWS/ETS expression in both animal and hMSCs emerged in parallel with effective and reproducible techniques to isolate and characterize these cell types.

### Murine MSC models

Ectopic expression of EWS/FLI in murine BM-MSCs was first described by Torchia et al. (2003) and unlike previous reports in human fibroblasts (Lessnick et al., 2002) stable EWS/FLI expression did not result in growth arrest or senescence. Furthermore, it was demonstrated that osteogenic and adipogenic differentiation was also impeded by EWS/FLI expression. Notably, the cultured BM-MSC came from INK4A/ARF deficient mice and no phenotypic assays of transformation were assessed such as proliferation assays, anchorage independent growth or xenograft tumor formation. However, these initial results importantly demonstrated tolerable EWS/FLI expression in murine MSCs and the capability of EWS/FLI to repress lineage-specific differentiation. In a similar series of experiments using a murine myoblast cell line (C2C12), which is capable of osteogenic, myogenic, and adipogenic differentiation, Eliazer and colleagues (2003) demonstrated a comparable tolerance of ectopic EWS/FLI expression and impaired terminal differentiation into muscle and bone. Similar to the Torchia et al. model, *in vitro* and *in vivo* assays testing transformation potential of these cells was not assessed.

A more comprehensive look at ectopic EWS/FLI expression in murine MSCs was later reported by Castillero-Trejo et al. (2005) and Riggi et al. (2005). Interestingly, both EWS/FLI-expressing MSC models were capable of forming tumors in immunodeficient mice. In the Riggi et al. study, subcutaneously injected EWS/FLI-expressing MSCs developed detectable tumors within 6 weeks. Histologically, these tumors appeared as sheets of small round blue cells and stained positive for common Ewing sarcoma cell surface markers, CD99, and NSE (Kovar et al., 1990; Ambros et al., 1991; Amann et al., 1999). Furthermore, these transformed MSCs maintained wild type p16^INK4A^/p19^ARF^ and a functional p53. Dysregulation of *IGF-1* and *TGFβRII* gene expression, two commonly up- and down-regulated targets in Ewing sarcoma, respectively, were also observed (Prieur et al., 2004; Smith et al., 2006). Importantly, these effects of EWS/FLI expression were not observed in murine embryonic stem cells. However, CD99 expression in this murine model has been since questioned as the immunoreactive extracellular domain of murine CD99 is highly divergent from humans (Kovar and Bernard, 2006). Using a slightly different approach, Castillero-Trejo et al. (2005) obtained stable EWS/FLI expression in MSCs procured from young mice (3–4 weeks). Using intravenous, intra-peritoneal or subcutaneous routes of administration, EWS/FLI-expressing MSCs rapidly developed into tumors in syngeneic mice. It was also noted that the efficiency of tumor induction was positively correlated with an increasing number of cell passages. Early passage and late passaged cells demonstrated a constitutively high expression of p53, while p21 expression (an up-regulated target of p53) gradually decreased with increasing cell passage. Acquired mutations in p53 were observed in some but not all of these late passage cells, however, using a Cre-transduced loxP-EWS/FLI construct, it was shown that MSC transformation is still highly EWS/FLI-dependent in early and late passage cells. Unfortunately, a comprehensive Ewing sarcoma gene expression profile was not documented in either study, questioning whether these transformed MSCs were definitively Ewing sarcoma.

A major concern using mouse-derived cells to model Ewing sarcoma is that Ewing sarcoma appears to be exclusively a human disease; spontaneous Ewing sarcomas have not been reported in other animal species. Additionally, the gene expression profiles of experimental “Ewing sarcoma-like” cells derived from murine NIH3T3 cells differ greatly from that observed in humans (Braunreiter et al., 2006). For example, two consistently up-regulated gene targets necessary for transformation in human-derived Ewing sarcoma cell lines, *NR0B1*, and *NKX2.2* are not dysregulated in murine-derived cells (Owen and Lessnick, 2006; Gangwal et al., 2008). It is known that EWS/FLI and related EWS/ETS fusions up-regulate numerous target genes via a promoter-based GGAA microsatellite response element (Gangwal et al., 2008, 2010; Guillon et al., 2009). These GGAA microsatellites are essential for EWS/ETS DNA binding and gene activation, yet these elements are absent in the murine orthologs of key up-regulated targets necessary for oncogenic transformation, such as *NR0B1, CAV1*, and *GSTM4* (Tirado et al., 2006; Gangwal et al., 2008; Luo et al., 2009). Another concern is that murine-derived MSC expanded in culture have also been shown to spontaneously transform in as few as three passages, attributable to spontaneous chromosomal translocations and genomic instability (Miura et al., 2006; Zhou et al., 2006). Human cells are generally regarded as more challenging to transform than their mouse counterparts, which are easily transformed and produce seemingly more aggressive tumors (Akagi, 2004).

### Human MSC models

Given these aforementioned limitations of murine MSCs and the propensity of Ewing sarcoma to develop exclusively in humans, inducible models utilizing human-derived MSCs intuitively provide a better *in vitro* model of histogenesis and EWS/FLI-mediated transcriptional regulation. Despite numerous ethical, cost, and access-related challenges associated with human MSC isolation and manipulation, numerous reports utilizing these cell types have emerged. Riggi and colleagues (2008), using similar techniques to their earlier work with murine MSCs, obtained stable expression of EWS/FLI in human bone derived-MSCs (hMSC^EWS/FLI^). Cell populations were isolated from a more “aged” adult population undergoing hip and knee replacement surgeries. Ectopic EWS/FLI expression in these cells resulted in typical morphological changes (cellular rounding and increased nuclear to cytoplasmic ratio) and profound up-regulation of CD99. It was noted that control hMSCs had a low basal expression of CD99. These morphological changes were accompanied by a modified gene expression profile highly concordant with public data sets from established Ewing sarcoma tumors and cell lines. When tested against a publicly available database of bone and soft tissue sarcomas (Baird et al., 2005), the experimental hMSC^EWS/FLI^ gene set most closely matched Ewing sarcoma, with an observed overlap of 40 (*p* < 0.00001). Classic EWS/FLI target genes such as *NKX2.2*, *NR0B1*, *IGF1*, *COL11A1*, *MMP1*, and *EZH2* were also up-regulated in this data set. EWS/FLI expression also induced numerous genes encoding neuroectodermal markers or neuronal differentiation pathways such as *NYP1R*, *GRP, MSX1, ERG2, NKX2.2, NGFR*, and *SOX2*. Interestingly, the life span and proliferation characteristics of the hMSC^EWS/FLI^ population were unchanged from control hMSC populations, which had a life expectancy of only 3 months. Additionally, in sharp contrast to previous studies in murine-derived MSCs, the hMSC^EWS/FLI^ cells maintained tri-lineage differentiation capabilities into bone, fat and cartilage. Despite the compelling gene expression characteristics hMSC^EWS/FLI^, tumors failed to develop when injected into the subcapsular renal component of immunocompromised mice.

Using a slightly different approach, Miyagawa and colleagues (2008), developed a tetracycline-inducible model of EWS/FLI expression in a human MSC line, UET13. Uniquely, this cell line is immortalized using human telomerase (hTERT) and E7 viral transgenes, while maintaining tri-lineage differentiation abilities. UET13^EWS/FLI^ cells demonstrated morphological changes and CD99 up-regulation comparable to Riggi et al. (2008), however, they observed an apoptosis-independent growth inhibition of induced cells. Ectopic EWS/FLI expression also resulted in cell surface immunophenotypes and gene expression profiles consistent with Ewing sarcoma, although no *in vitro* or *in vivo* measures of transformation were assessed.

After the same group recently reported CD133+ cancer stem cell (CSC) subpopulations in dissociated primary Ewing sarcoma tumors (Suva et al., 2009), Riggi et al. (2010) tested the hypothesis that similar CD133+ CSC populations could be identified using their previously described hMSC^EWS/FLI^ model. Using a slightly different approach, they manipulated two distinct hMSC populations: hMSCs isolated from an older adult population and a younger pediatric population ranging in age from 6 to 14 years old. They observed similar mRNA and protein levels of EWS/FLI, cell surface immunophenotypes and tri-linage differentiation capabilities between adult and pediatric hMSCs^EWS/FLI^ but noted a markedly increased proliferation rate in the pediatric-derived cells. Gene expression profiles were also dissimilar between the two groups: pediatric hMSCs^EWS/FLI^ induced greater expression of common EWS/FLI targets, *NKX2.2, IGF1, SOX2, NPY1R, GRP*, and *EZH2* and exhibited a greater gene overlap with the Baird et al. dataset of bone and soft tissue sarcomas (Baird et al., 2005) when compared to adult hMSCs^EWS/FLI^. This overlapping Ewing sarcoma molecular signature was even more pronounced when pediatric hMSCs^EWS/FLI^ were expanded in a serum-free media. Serum-starved growth conditions have been shown to facilitate genetic reprograming of differentiated human and murine cells into induced pluripotent stem cells (iPS) (reviewed in Jaenisch and Young, 2008). Using these conditions, pediatric hMSCs^EWS/FLI^ strongly up-regulated numerous genes associated embryonic stem cells such as *OCT4, SOX2, NANOG*, and *C-MYC*. *OCT4* and *NANOG* were not expressed in adult hMSCs^EWS/FLI^ and expression of these stem cell-associated targets increased 20-fold in serum starved pediatric hMSCs^EWS/FLI^.

As a previously established cell surface marker of Ewing sarcoma CSCs, CD133 expression was scarce in adult hMSCs^EWS/FLI^ but increased 15-fold and 250-fold in pediatric hMSCs^EWS/FLI^ expanded in normal and serum-free conditions, respectively. These data interestingly demonstrate phenotypic differences in adult and pediatric hMSCs populations expressing EWS/FLI, suggestive that cells from pediatric sources have a greater proliferative ability, have a EWS/FLI-mediated transcriptome most similar to Ewing sarcoma tumors and cells lines and furthermore, are more likely to show evidence of genetic reprogramming toward a more pluripotent, stem cell phenotype. Unfortunately, similar to their preceding work, *in vitro* and *in vivo* transformation capabilities of these more “Ewing sarcoma-like” pediatric hMSCs^EWS/FLI^ were not assessed and/or reported.

Data from murine and human MSC models of Ewing sarcoma have demonstrated that these progenitor cells afford a permissive or compatible cellular environment, tolerant of EWS/FLI expression with minimal toxicity. Changes in cell morphology, CD99 up-regulation, neuroectodermal characteristics, repressed differentiation status and global gene expression profiles are all strikingly similar to profiles of Ewing sarcoma cell lines and primary tumors. Despite these common features, ectopic expression in hMSCs does not fully recapitulate oncogenic transformation. Robust xenograft tumor development in murine-derived MSCs expressing EWS/FLI challenge this assertion, however, evidence obtained by modeling Ewing sarcoma, an exclusively human malignancy in human cells justifiably takes scientific precedence over non-human model systems at this point in time. Synthesizing this information it is possible that MSCs merely provide a cellular environment tolerant of the EWS/FLI oncoprotein and the aforementioned genetic and phenotypic alterations observed in MSCs^EWS/FLI^ are simply a manifestation of the EWS/FLI transcriptional signature and the true cell of origin remains elusive. Conversely, MSCs may indeed be the most probable cell of origin, however other crucial genetic alterations either up- or down-stream of the principal defining EWS/ETS translocation event, cooperatively necessary for malignant transformation have yet to be identified. These challenges are further appreciated when considering the various attempts to develop a transgenic mouse model of Ewing sarcoma: constitutively expressed EWS/FLI is embryonic lethal (Torchia et al., 2007), where inducible, tissue-specific expression of EWS/FLI in MPC resulted in limb bud developmental abnormalities (Lin et al., 2008). Although somewhat encouraging, using this same transgenic model in a p53 deficient background resulted in poorly differentiated sarcomas (Lin et al., 2008).

Irrespective of our evolving understanding of molecular paradigm of transformation in Ewing sarcoma, MSC have proven to be a reliable, readily obtainable, scientific tool and will have numerous applications moving forward. hMSCs can be obtained from a variety of adult, pediatric and post-natal tissues and cultured using various techniques. It is becoming increasingly apparent that these subtle differences greatly influence down-stream applications. Consequently, understanding these complexities is important, as these progenitor cells are becoming an increasingly useful platform to answer the principal questions proposed above.

## Mesenchymal stem cell basics

### Discovery

The osteogenic properties of bone marrow elements have been understood dating back to the 19th century, although it was only 50 years ago when Friedenstein and colleagues identified a sub-population of non-hematopoietic bone marrow-derived cells capable of osteogenic differentiation (Owen and Friedenstein, 1988). Initially coined “osteogenic stem cells,” these cells (or highly related populations) have since been shown capable of multi-lineage differentiation into fat, muscle, bone, cartilage, tendon, fibrous tissue, and were subsequently re-assigned the title of “mesenchymal stem cells” in the late 90's (Dennis and Caplan, 1996; Pittenger et al., 1999). Since then, these pluripotent cells have adopted numerous titles such as “multi-potent mesenchymal progenitor cells,” “mesenchymal stromal cells,” “skeletal stem cells” and so forth. Existing in an uncommitted state, under the appropriate signaling environment these cells posses reproducible lineage-specific regenerative abilities and more recently, have been shown to modulate elements of the immune system and are being intensively developed for tissue regeneration purposes (reviewed in Chamberlain et al., 2007). Consequently, scientific inquisition into the finer biological attributes of these cells has gained considerable traction over the past two decades.

### MSC isolation

The warehouse of mesenchymal stem cells was traditionally viewed as the stromal component of bone marrow, largely in part to the origins of cell discovery in this location. Subsequently, a vast array of anatomic sites have been recognized as a reliable deposit of these cells and includes compact bone, adipose tissue, peripheral blood, and placental tissues such as the umbilical cord, umbilical cord blood (UCB), the placenta, and amniotic fluid (Wagner et al., 2005; Riggi et al., 2008; Hass et al., 2011). hMSCs are commonly isolated from the mononuclear cell layer of the aforementioned tissues after cell dissociation and density gradient centrifugation. Various pre- and post-centrifugation techniques have been described to enrich the mononuclear cell layer with likely MSC populations including hypotonic red blood cell lysis buffers, negative selection antibody beads (removing hematopoietic stem cells) and cell sorting using a FACS or MACS-type systems. Culture conditions for the mononuclear cell extract are extremely variable (Wagner et al., 2005; Miyagawa et al., 2008; Riggi et al., 2008; Secco et al., 2008; Malgieri et al., 2010; Peters et al., 2010), but commonly include a minimal growth medium such as Minimal Essential Medium (MEMα) or Dulbecco's Modified Eagle's Medium (DMEM), supplemented with 10% fetal bovine serum, antibiotics, and antifungals. In a study by Sotiropoulou et al. (2006), use of MEMα media supplemented with l-glutamine (MEMα-LG) or glutamax (MEMα-GL) afforded the most optimal MSC growth conditions. Various recombinant growth factor such as fibroblast growth factor basic (bFBF), epidermal growth factor (EGF) and platelet derived growth factor-BB (PDGF-BB) are potent mitogens, prolonging the proliferative life span of MSCs and are important medium supplements for rapid cellular expansion, while maintaining a non-committed state (Lennon et al., 1995; Tamama et al., 2006; Ng et al., 2008). However, there is concern that these growth factor supplements may direct subtle and often unwanted lineage commitment. For example, the most common growth factor additive to MSC media, bFGF has been shown to favor differentiation toward osteogenic lineages and suppresses neurogenic potential (Martin et al., 1997; Sotiropoulou et al., 2006). Mononuclear cells are then expanded on tissue culture plastic and after 3–4 days of culture, non-adherent cells, most of which are of hematopoietic origin are discarded. MSCs comprise the adherent cell population in addition to some hematopoietic progenitor cells, which are progressively washed away over time. Growth characteristics of early cultured MSC are characterized by an initially lag phase lasting 3–4 days, followed by more rapid proliferation phase with population doubling times highly dependent on the anatomic site/fluid of procurement and the age of the subject (D'Ippolito et al., 1999; Stenderup et al., 2003; Baksh et al., 2007; Hass et al., 2011).

### MSC characterization and phenotypic heterogeneity

Phenotypically, MSCs express a variety of cell surface markers, although none are entirely MSC-specific. It is agreed upon that MSC lack the expression of hematopoietic markers such as CD45, CD34, CD14, or CD11 and the endothelial marker CD31, but generally express, CD44, CD71, CD73, CD90, CD105, Stro-1, and adhesion molecules such as vascular cell adhesion molecule (VCAM-1), intercellular adhesion molecule (ICAM-1), activated leukocyte cell adhesion molecule (ALCAM) and CD29. The constellation of these expressed surface markers does vary depending on the tissue source, isolation and culture techniques and population doublings (comprehensively reviewed in Hass et al., 2011; Chamberlain et al., 2007; Malgieri et al., 2010). Regardless, a precise panel of cell surface markers specific to MSCs, let alone tissue-specific MSCs, has not been established.

Consequently, perhaps the most important defining attribute of presumed mesenchymal stem cell populations is functional. As presumed precursors of mesenchymal tissue, tri-lineage differentiation potential of these cells into fat, bone, and cartilage is a vital identifying characteristic of MSCs. These multi-potent characteristics are routinely assessed under *in vitro* conditions: osteogenic differentiation is achieved by exposing cell monolayers to ascorbic acid, β-glycerophosphate and dexamethasone. Differentiation is identified using alizarin red and von Kossa stains. Chondrogenic differentiation is promoted by centrifuging cells into pellets and exposing these masses to transforming growth factor beta (TGF-β). Strong toluidine blue staining and expression of collagen type II are indicative of chondroid differentiation. Lastly, adipogenic differentiation is achieved by exposing cell monolayers to insulin, dexamethasone, isobutyl-methly-xanthine, and indomethacin. Lipid rich vacuoles within cells are easily identified by oil red O staining (Mackay et al., 1998; Pittenger et al., 1999; Lee et al., 2004; Dominici et al., 2006; Secco et al., 2008).

It is now generally accepted that cells expressing and lacking a consistent core signature of MSC cell surface markers, in addition to inducible tri-lineage differentiation into bone, cartilage and fat adequately define a mesenchymal stem cell population. In keeping with this, the International Society for Cellular Therapy (ISCT) has established a minimal set of guidelines used to characterize and define “multipotent mesenchymal stromal cells,” also abbreviated MSC. Using these guidelines, presumptive MSC populations must possess the following phenotypic features: adherence to tissue culture plastic, expression of surface markers CD73/CD90/CD105, absent expression of CD34/CD45/CD14 or CD11b/CD79α or CD19/HLA-DR, and tri-lineage differentiation into osteoblasts, chondroblasts, and adipocytes (Dominici et al., 2006). Table 1 summarizes the ISCT guidelines and described phenotypic differences between human MSC from various tissue sources.

**Table 1:** **hMSC sources and biological characteristics**.

	**Positive cell surface antigens**	**Negative cell surface antigens**	**Tri-lineage potential**	**Isolation frequency**	**Self renewal potential**
ISCT guidelines for “Multipotent mesenchymal stromal cells” (MSC)	>95% cells: CD73 (SH3/SH4), CD90 (Thy-1), CD105 (SH2)	<2% cells: CD34, CD45, CD14 or CD11b, CD79 or CD19, HLA-DR	Osteogenic Adipogenic Chondrogenic	n/a	Unspecified
Bone marrow-derived MSC (BM-MSC)	CD13, CD44, CD73, CD90, CD105, CD166, STRO-1	CD14, CD31, CD34, CD45, HLA-DR	Osteogenic Adipogenic Chondrogenic ± Neurogenic	1/10^5^–10^6^ MNCs 100% isolation efficiency	Susceptible to contact inhibition Doubling time = 40–60 h Senescence 10–30 PD
Adipose tissue-derived MSC (AT-MSC)	CD9, CD13, CD29, CD44, CD54, CD73, CD90, CD105, CD106, CD146, CD166, STRO-1	CD11b, CD14, CD19, CD31, CD34, CD45, CD79, CD133, CD144	Osteogenic Adipogenic Chondrogenic ± Neurogenic	1/10^4^–10^5^ MNCs 100% isolation efficiency	Susceptible to contacy inhibition Doubling time = 45 h Senescence 10–30 PD
Umbilical cord blood derived MSC (UC-MSC)	CD29, CD44, CD73, CD90, CD105	CD19, CD31, CD33, CD34, CD45, CD90, CD117, CD133, CD135, HLA-DR	Osteogenic Chondrogenic Neurogenic Adipogenic	1/10^7^–10^8^ MNCs 30–70% isolation efficiency	Resistant to contact inhibition Doubling time = 24 h Senescence 30–80 PD

## Salient features of human MSCs—implications for ewing sarcoma modeling

### hMSC proliferation and differentiation capabilities

In the most basic terminology, “stem cells” are commonly regarded as undifferentiated precursor cells capable to proliferation, self-renewal and differentiation into multiple lineage-specific progeny (Blau et al., 2001). Based on *in vitro* behavior, MSCs possess many of these attributes however, MSCs do not possess an unlimited proliferative potential and the differentiation potential of MSCs lessens with increasing cell passages. For example, BM-MSCs, show phenotypic and molecular evidence of senescence after 10–30 population doublings, which is also associated with decreased osteogenic and chondrogenic differentiation capabilities (Colter et al., 2000; Chen et al., 2012). Low-density seeding of hMSCs has been shown to increase cell proliferation rates and life expectancy, although to a limited extent (Colter et al., 2000). Interestingly, adipogenic differentiation appears to be less affected by cell passages (Chen et al., 2012). In the Riggi et al. model of hMSC^EWS/FLI^, both control and experimental MSCs stopped proliferating at 3 months (Riggi et al.). The heterogeneous cellular constitution of most density gradient-derived mononuclear cell isolates often necessitates numerous cell passages to remove contaminating adherent hematopoietic cells, potentially diminishing the “stem cell capabilities” of harvested MSCs.

To circumvent these issues, immortalizing cells with retroviral hTERT, SV40 T-antigen and E7 transgenes can substantially prolong the proliferative life span of hMSCs without loss of tri-lineage potential (Terai et al., 2005; Wolbank et al., 2009; Li et al., 2010). There is, however, discrepancy in the literature as to whether telomerase immortalized hMSCs can transform over time. In one study, after 256 population doublings, the hTERT-transduced MSC line, hMSC-TERT20 was shown to spontaneously transform *in vitro* and in xenograft transplants (Serakinci et al., 2004), whereas other studies have failed to demonstrate any transformation abilities in these immortalized cell types (Terai et al., 2005; Wolbank et al., 2009). As described earlier, ectopic expression of EWS/FLI in an immortalized hMSC line (UET-13) has been investigated, although *in vitro* and *in vivo* transformation abilities of these cells were not assessed and/or reported (Miyagawa et al., 2008). Certainly for the purposes of *in vitro* modeling of Ewing sarcoma, immortalization of hMSCs can be easily achieved, is unlikely to affect transformation under 100 PDs and may greatly increase the window of opportunity to select a homogenous population of MSCs without losing the stem cell phenotype.

### Age-related biological attributes of MSCs

In regards to the biological potential of animal and hMSCs, there is no doubt that age matters. It has been shown that the number of hMSCs isolated from bone marrow is greatest at birth and steadily decreases with increasing age (Caplan, 2009). Furthermore, proliferation rates, life expectancy, differentiation capabilities and contact inhibition are markedly reduced in human and animal MSCs harvested from older subjects (D'Ippolito et al., 1999; Stenderup et al., 2003; Hass et al., 2011; Chen et al., 2012). Liposomal transfection rates of hMSCs are also negatively affected by an aging source (Baksh et al., 2007). Given that Ewing sarcoma is primarily a pediatric disease, Riggi et al. (2010) compared ectopic EWS/FLI expression in pediatric and adult-derived MSCs. Pediatric MSCs had a greater proliferation rate, shared more molecular similarities to Ewing sarcoma and had greater expression of NCSC and Ewing sarcoma CSC markers compared to their adult counterparts. Explanations for these findings remain unresolved, but it is clear there are intrinsic differences between adult and pediatric/neonatal hMSCs, which may provide a more accessible molecular environment for EWS/FLI-mediated transcriptional regulation. It should be noted that pediatric hMSCs^EWS/FLI^ have not been shown to transform *in vitro* or *in vivo* suggesting the age-related molecular biology of these cells is unlikely the missing genetic alteration required for EWS/FLI-mediated transformation.

### Tissue-specific attributes of hMSCs

Since their discovery, MSCs have been successfully isolated from a diverse spectrum of human tissues and fluids. Presently, the most common sources of MSCs are bone marrow, compact bone, adipose tissue, and placental tissues such as umbilical cord and UCB (Riggi et al., 2005; Kern et al., 2006; Malgieri et al., 2010; Hass et al., 2011). The frequency of MSCs in each of these tissues and the success at which these cells can be cultivated varies considerably (Table 1). Adipose tissue is the most abundant source of MSCs, followed closely by bone marrow, whereas MSCs are relative scarce in UCB and peripheral blood (Pittenger et al., 1999; Kern et al., 2006; Rebelatto et al., 2008). These numbers likely contribute to the high success of MSC isolation from bone marrow and adipose tissue and the inconsistent numbers reported for UCB (Wagner et al., 2005; Kern et al., 2006; Hass et al., 2011). Functionally, it is generally accepted that irrespective of the tissue source, MSCs exhibit relatively consistent tri-lineage differentiation potential.

Culturing and expanding placenta-derived MSCs has gained increasing traction for numerous reasons: unlike BM-MSCs and AT-MSCs, procurement of placental-derived MSCs is not painful, invasive or associated with procedural complications. Placental tissues are regarded as medical waste and are in abundant supply, therefore a plentiful source of MSCs. Placentas are a neonatal tissue and therefore provide a MSC population with more desirable “young” growth characteristics and life expectancy (Kern et al., 2006; Lu et al., 2006; Baksh et al., 2007). Moreover, MSCs from UCB are more primitive and exhibit greater pluripotent plasticity than MSCs isolated from adult tissues (Sarugaser et al., 2005; Lu et al., 2006). Ewing sarcoma models have not been attempted in UCB-MSCs, although the favorable biological attributes of these MSCs may provide a better age-related biological platform for future studies. There are some drawbacks of UCB-MSCs, namely UCB is also a source of adherent endothelial progenitors cells, which may contaminate adherent MSC populations (Broxmeyer et al., 2006). Although, CD31 is a reliable endothelial marker and cell-sorting techniques could be used to circumvent this problem. Additionally, roughly 15–20% of UCB samples are contaminated with low numbers of maternal blood cells (0.04–5.0%) (Hall et al., 1995; Tsang et al., 2002). However, given the paucity of MSCs in peripheral blood, the mesenchymal progenitor burden of this maternal contamination would be exceeding low.

## Future directions and conclusions

Attempts to delineate the precise cell of origin in Ewing sarcoma have demonstrated that MSCs can indeed be used as a functional molecular vehicle to investigate the consequences of EWS/FLI expression in a native cellular context. Modeling Ewing sarcoma using MSCs will be an important strategy moving forward; profiling genetic differences between incompletely transformed MSCs and Ewing sarcoma tumors and cell lines may help identify the elusive genetic perturbations required for transformation. EWS/FLI-expressing hMSCs may also facilitate a greater understanding of how EWS/ETS chimeras interact with DNA elements and cooperative proteins in a more native cellular environment. However, the intricacies hMSC isolation, identification and experimental manipulation have greatly evolved overtime. Consequently, effective use of these multipotent stem cells requires a more detailed understanding of the salient age-, tissue- and culture-associated aspects of MSC biology. Utilizing pediatric or neonatal tissue sources in combination with transgenic methods of immortalization may ultimately improve accessibility and passage-related hurdles, while potentially providing a more representative, pluripotent cell population implicated in Ewing sarcoma.

### Conflict of interest statement

The authors declare that the research was conducted in the absence of any commercial or financial relationships that could be construed as a potential conflict of interest.
